# The Role of Regulatory T Cells in Pulmonary Arterial Hypertension

**DOI:** 10.3389/fimmu.2021.684657

**Published:** 2021-08-19

**Authors:** Wen Tian, Shirley Y. Jiang, Xinguo Jiang, Rasa Tamosiuniene, Dongeon Kim, Torrey Guan, Siham Arsalane, Shravani Pasupneti, Norbert F. Voelkel, Qizhi Tang, Mark R. Nicolls

**Affiliations:** ^1^Department of Medicine, VA Palo Alto Health Care System, Palo Alto, CA, United States; ^2^Department of Medicine, Stanford University School of Medicine, Stanford, CA, United States; ^3^Department of Pulmonary Medicine, Amsterdam University Medical Centers, Amsterdam, Netherlands; ^4^Department of Surgery, University of California San Francisco, San Francisco, CA, United States

**Keywords:** regulatory T cell, pulmonary arterial hypertension, sexual dimorphism, right ventricle, estrogen

## Abstract

Pulmonary arterial hypertension (PAH) is a chronic, incurable condition characterized by pulmonary vascular remodeling, perivascular inflammation, and right heart failure. Regulatory T cells (Tregs) stave off autoimmunity, and there is increasing evidence for their compromised activity in the inflammatory milieu of PAH. Abnormal Treg function is strongly correlated with a predisposition to PAH in animals and patients. Athymic Treg-depleted rats treated with SU5416, an agent causing pulmonary vascular injury, develop PAH, which is prevented by infusing missing CD4^+^CD25^high^FOXP3^+^ Tregs. Abnormal Treg activity may also explain why PAH disproportionately affects women more than men. This mini review focuses on the role of Tregs in PAH with a special view to sexual dimorphism and the future promise of Treg therapy.

## Introduction

The first classification of pulmonary hypertension (PH) was proposed in 1973 and is now defined as an increase in mean pulmonary arterial pressure (mPAP) > 20 mmHg at rest, normal left atrial pressure and pulmonary vascular resistance >= 3 Wood units (1). This review focuses on PAH which is classified by World Health Organization (WHO) as Group 1 PH (1). PAH includes idiopathic or sporadic cases (IPAH), heritable cases (HPAH, describing patients with a family history or identified germline mutations), and associated forms (attributable to conditions like anorexigens, liver diseases, congenital heart diseases, and connective tissue diseases) (2). Heterozygous germline mutations in gene encoding for bone morphogenetic protein receptor 2 (BMPR2) account for 53-86% of the familial cases of PAH and 14-35% of patients with IPAH (3). Although clinical features of HPAH and IPAH are indistinguishable, patients with HPAH have an earlier disease onset with more severe hemodynamics (4). There is a strong sex-associated influence on the development of PAH, with an approximate female to male patients’ ratio of 4:1 (5). Although the incidence of PAH is higher in women, the estimated 5-year survival is less favorable in men (52% in men compared to 62% in women) (6). Regardless of the underlying etiology, patients with PAH exhibit similar pathological alterations including remodeling of the pulmonary arterioles driven by proliferation/dysfunction of both pulmonary arterial endothelial cells (PAECs) and smooth muscle cells (PASMCs), *in situ* thrombosis, increased pulmonary arteriole contractility, and enhanced inflammatory infiltrates (7–9).

PAH is a lethal cardiopulmonary condition characterized by pulmonary vascular remodeling and right heart failure (10). Drugs used to treat this disease primarily target pathologic vasoconstriction, but there is an additional rationale for treating immune dysregulation (9). Growing evidence indicates that abnormal Treg activity promotes the development of PAH, and accentuating the activity of these cells has therapeutic potential (9). Tregs play a vital role in maintaining immune homeostasis, fostering tissue regeneration, and limiting vascular injury (11–13). They protect against autoimmunity by dampening inappropriate responses to self-antigen (14). This defensive activity was first identified in mice lacking thymic-derived CD4^+^CD25^hi^Forkhead box P3 (FOXP3)^+^ Treg cells and in Treg-depleted animals which develop a constellation of autoimmune conditions including thyroiditis, diabetes, autoimmune gastritis, and inflammatory bowel disease (14, 15). Subsequently, numerous pre-clinical autoimmunity models show that Treg defects are implicated in the development of autoimmune diseases and prevented by the adoptive transfer of Tregs (14). Other types of CD4^+^ regulatory T cells are also discovered including IL-10-producing type 1 regulatory T cells and TGF-β producing-T helper 3 cells (16, 17). Here, we only discuss the CD25^high^FOXP3^+^ Treg population given the broader and indispensable roles of these cells.

PAH-associated conditions exhibit Treg abnormalities (18). Furthermore, Treg dysfunction in PAH affects males and females differently and may arise because of pathogenic gene variants (e.g., mutations in *BMPR2* and chronic vascular inflammation (18–20). Our group demonstrated that adoptive transfer of Tregs is effective in preventing pre-clinical PAH (21). Treg infusions restore immune regulation, reduce endothelial injury, impede PAH-mediated vascular remodeling, and prevent right heart failure (21, 22). Because Tregs can limit and potentially reverse pulmonary vascular disease, Treg infusions may become a viable treatment for this condition (23). This review explores how genetic and environmental cues may cause Treg abnormalities that exacerbate PAH and discusses how the restoration of Treg function may attenuate this life-threatening disease.

## Regulation of Treg Ontogeny, Identity, and Function

Anti-inflammatory CD4^+^CD25^high^FOXP3^+^ Tregs comprise approximately 5% of CD4^+^ T cells and develop primarily in the thymus (24–27). Genetic mutations (e.g., *FOXP3*, *RAG1*, and *AIRE*) (28) impact Treg thymic development and predispose individuals to autoimmune conditions. Continued expression of FOXP3 and sustained FOXP3 signaling are required for Tregs to maintain their lineage-stability and function, and FOXP3 expression is influenced by local tissue microenvironment and sex hormones (26). For example, FOXP3 may be turned on in conventional T cells (Tconvs, CD4^+^FOXP3^-^) at peripheral inflammatory sites to promote Treg phenotype (pTregs) (29). High concentrations of interleukin-1 beta (IL-1β) as well as interleukin-6 (IL-6) may result in decreased FOXP3 and increased interleukin-17 (IL-17) expression in Tregs (exTregs) (30–32). Specifically, proinflammatory IL-6, in conjunction with IL-1 and IL-23, induces the expression of RORγt and IL-17 and suppresses FOXP3 thereby causing a genetic reprograming in FOXP3^+^ Treg cells (33). Elevated IL-6 also promotes the methylation of a conserved Cp-G-rich island of *FOXP3* gene and results in reduced gene transcription (34). Notably, IL-1β, IL-6, and IL-17 are elevated in PAH and contribute to Treg instability and pathogenic inflammation (35, 36). Additionally, Tregs can specialize into T helper-(Th-) like subsets expressing Th1, Th17, Th2, or Th22 markers and receptors that mimic and suppress Th1, Th17, Th2, or Th22 immunity, respectively (37).

Abnormalities in thymic development and Treg production may be related to the most common genetic mutations in PAH involving *BMPR2* (38). Normal thymus development requires bone morphogenic protein (BMP) signaling (39); thymic epithelial cell maturation requires BMP4 (a BMPR2 ligand) and the activation of *FOXN1* transcription factor (40). Consequently, *BMPR2* mutations may affect embryonic thymic BMP4 signaling and influence Treg development in early life. It is unknown whether patients with BMPR2 mutations have decreased Tregs or whether the impact of BMPR2 signaling may be more subtle, not affecting Treg numbers globally but rather subset percentages and function. In peripheral lymphoid tissues, BMPR1α sustains the expression of FOXP3 in pTregs and is required for the maturation and preservation of Treg cell phenotype while inhibiting the differentiation of pro-inflammatory Th1 and Th17 cells (41). Missense mutations of BMPR1β were identified in pediatric IPAH cases (42) suggesting that BMPR1β insufficiency may contribute to the imbalance between Treg and Th1/Th17 populations in PAH. Genetic and environmental factors that govern Treg development and functionality (43) may ultimately play a role in the predisposition to PAH after vascular injury.

## PAH and Treg Abnormalities

The immune dysregulation observed in PAH may be attributable to Treg anomalies (9, 18). Tregs maintain immune homeostasis, and their deficiency predisposes individuals to autoimmune injury (13, 26, 44, 45). Just as failing Tregs are increasingly recognized in cardiovascular diseases, abnormalities in Treg number and function are also reported in PAH (18, 36, 46–49). Inflammatory pathologies are associated with poor clinical outcomes in PAH and underscore the importance of understanding Treg derangements (9). Extensive pre-clinical evidence from our group and others suggest that abnormal Treg activity may explain autoimmune and inflammatory features noted in PAH (**Table 1**). Treg numbers are reduced in PAH lungs but increased in the peripheral circulation, displaying reduced suppressive functionality (46, 47, 77, 78). In addition to the thymic anomalies, defective leptin and adiponectin signaling may also influence Treg function in IPAH patients (46, 79).

**Table 1:** Clinical and pre-clinical studies show a relationship between Treg abnormalities and PAH-associated conditions.

	PAH-associated conditions	PAH prevalence	Treg (T cell) abnormalities
**Clinical Studies**	Scleroderma	≈10% (50)	↓Treg function and frequency (51)
	Lupus erythematosus	3%-23% (52)	Altered Treg frequency, ↓Treg/Teff(effector T cells) ratio correlate with disease severity (53)
	Sjögren’s syndrome	≈10% (54)	Altered Treg frequency; ↓Treg/Th17 (55)
	Polymyositis	≈10% (56)	↓Treg frequency and ↓Treg/Teff ratio (57)
	Antiphospholipid syndrome	Common (58)	↓Treg frequency and ↓Treg/Teff ratio (59)
	Hashimoto’s thyroiditis	case reports (60)	↓Treg/Teff ratio and ↓Treg function (61)
	HIV infection	0.5% (62)	Altered Treg frequency, phenotype, and function (63)
	Schistosomiasis	8%-25% (64)	Altered Treg frequency (65)
	Herpesvirus 8	≈3% (66)	Altered Treg frequency and function (67)
	DiGeorge syndrome	case reports (68, 69)	Abnormal thymic Treg development (70)
	IPEX syndrome	case report (ATS) (71)	FOXP3 mutations and Treg defects (72)
	APS-1	case report (73)	AIRE mutations and Treg defects (74)
	Idiopathic PAH	n/a	↓Treg/Th17 (75)
	CTD-PAH	n/a	↓Treg frequency, ↓Treg/Teff ratio and ↓Treg function (36)
**Pre-clinical**	Athymic rats	100% after SU5416	↓Treg causes PAH (w/o hypoxia), Treg reconstitution protects rats from PAH
	B6 mice	100% after hypoxia	Treg infusion protects mice against PAH (76)

The heterogeneous vascular lesions of PAH reveal numerous inflammatory cell types in and around the pulmonary vessels, suggesting a coordinated immune response (9, 80). Treg abnormalities may promote the development and progression of PAH by failing to quell this inflammation following vascular injuries, such as that induced by shear stress, hypoxia, ischemia, or pathogens; the result, a prolonged and destructive period of vascular wound healing (9, 18, 20, 21, 81, 82). Beyond the regulation of adaptive immunity, Tregs control innate responses following injury. In a Treg-deficient model of PAH, we show that the absence of Tregs contributes to the emergence of destructive macrophage-based immunity culpable in progressive endothelial damage and vascular remodeling (83). Tregs can also control neutrophilic infiltration and preserve endothelial barrier function, possibly relevant to the pathogenesis of PAH (84, 85).

## Modeling Treg Biology in PAH

Aberrant Treg activity predisposes animals to vascular inflammation and PAH (86). We initially noted a predisposition to severe PAH in animals lacking normal T cells (athymic rats) after exposure to SU5416, a vascular endothelial growth factor 2 (VEGF2) receptor antagonist that causes pulmonary arteriole injury. [Rats robustly model PAH in a manner not uniformly observed in mice (87)]. Treg-deficient rats exposed to SU5416 demonstrate an accumulation of B cells and macrophages after one week before developing hemodynamically-significant PAH (21). Restoring missing CD4^+^CD25^hi^FOXP3^+^ cells through intravenous infusion limits inflammation, prevents endothelial apoptosis, and ameliorates PAH in this model; these original studies demonstrate a relationship between Treg deficiency and the proclivity for PAH. In SU5416-treated athymic rats, activated macrophages expand and produce leukotriene B_4_ (LTB_4_) in a lung environment lacking Tregs. This eicosanoid may contribute to pulmonary vascular disease by inducing endothelial cell apoptosis/transformation, smooth muscle cell proliferation/hypertrophy, and adventitial fibroblast proliferation/migration (83, 88). Interestingly, unlike some animal models which observe PAH more consistently in males, the Treg-deficient rat model shows a profoundly heightened vulnerability to disease in both sexes. In the future, other PAH models (currently in development), with more subtle Treg deficits than the athymic rat, will provide greater opportunities to understand the unique participation of Tregs in pulmonary vascular health and disease.

## Tregs and Sex-Related Pathology in PAH

**Figure 1:**
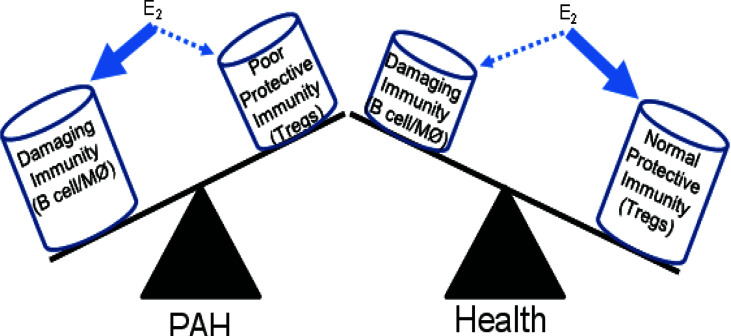
Differential effects of estrogen on immunity in PAH and health. Estrogen (E_2_) has complex effects on the immune response. In the absence of normal Treg immunity, E_2_ may promote damaging B cell and macrophage responses that induce pulmonary vascular disease and PAH. In health, the positive effects of E_2_ fostering Treg maturation may dominate.

PAH disproportionately affects women more than men (89). Our recent athymic rat study highlights the effect of sex on Treg activities in PAH and suggests the Tregs’ dominant role in protecting females against this condition (22). Treg sexual dimorphism is a complex subject. While males have a larger thymus, higher Treg numbers, and more robust Treg immune-suppressive capacity, female hormones enhance Tregs’ growth and function (90, 91). Because PAH is worse in females in this Treg-deficient model, estrogen (17b-estradiol; E_2_) may be exacerbating immunity in the absence of normal Treg function. E_2_ is a highly pleiotropic hormone for immune function, being both pro-inflammatory and anti-inflammatory under different conditions and with varying types of cells (92). E_2_ promotes Treg differentiation (93–96) while also enhancing Th2 responses (97) and B cell/macrophage activation (98–100). Treg suppressive activity involves E_2_-dependent expression of the anti-inflammatory checkpoint molecule Programmed Death-1 (PD-1) (96). Cumulatively, these findings raise the seemingly divergent possibilities that E_2_ may promote protective Treg function and that E_2_ can intensify harmful immunity (**Figure 1**). Because E_2_ supplementation may be a possible therapeutic strategy in PAH (84), it is crucial to discern the molecular underpinnings of E_2_ in PAH immune injury. Finally, PAH is prevalent in obese women (101). Tregs are decreased in adipose tissue, a phenomenon that may contribute to persistent low-grade inflammation. Reduced Tregs, expressing adiponectin receptor 1, in the lungs of obese mice promotes inflammation and a predisposition to PAH (79).

In the absence of Tregs, female PAH rat lungs exhibit worse hemodynamics, increased macrophage infiltration, a more significant decline in right ventricular (RV) capillary density, and greater RV perivascular and interstitial fibrosis (22). Treg infusion mitigates PAH and prevents these pathologic changes. Further, prostacyclin (PGI_2_), a potent vasodilator, is decreased in females with PAH and may also contribute to the sex-related differences. Following vascular injury and Treg therapy, PGI_2_ blood levels increase dramatically. Cyclooxygenase-2 (COX-2) and PGI_2_ synthase (PTGIS), critical enzymes for PGI_2_ synthesis, are profoundly upregulated in the lungs of Treg-treated rats. Blocking COX-2, heme oxygenase-1 (HO-1), and Programmed Death Ligand-1 (PD-L1)/PD-1 signaling pathways abrogate Treg protection from PAH (22). Thus, in the absence of regular Treg activity, reduced pulmonary COX-2/PTGIS expression and serum PGI_2_ levels correlate with worse disease in females. For these reasons, Treg therapy holds promise for PAH patients by helping them restore PGI_2_ production in their affected lung tissue. Presently, how Tregs promote PGI_2_ synthesis in the major endothelial cell production sites is unknown.

**Figure 2:**
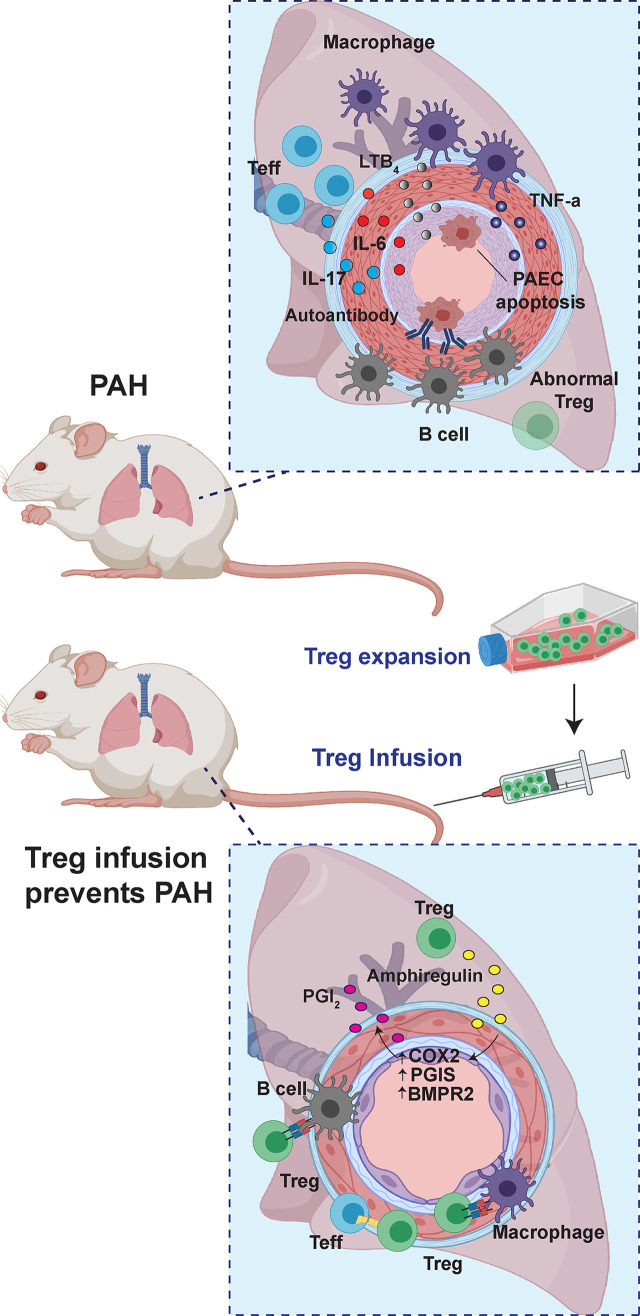
The role of Tregs in PAH. In Treg-deficient rats, vascular injury culminates in enhanced B cell and macrophage-dominated inflammation. With Treg infusion before the induction of vascular injury, PAH is avoided through upregulation of anti-inflammatory and vasoprotective pathways, which include upregulation of COX2, PGIS, and BMPR2.

Pulmonary arteries and RV capillaries remodel in evolving PAH (102). Treg infusion into T cell-deficient rats, treated with SU5416, do not develop pulmonary vascular disease and show increased expression of COX-2, PTGIS, HO-1, and PD-L1 in the smooth muscle cell layer of pulmonary arterioles. The Treg-protected RV, by distinction, demonstrates increased expression of these protective molecules in cardiac intimal cells and myocardium (22). Treg therapy may additionally afford protection in PAH by upregulating vascular wall BMPR2 (**Figure 2**). *In vitro*, Tregs cocultured with cardiac endothelial cells increase the expression of COX-2, PTGIS, HO-1, PD-L1, PGI_2_, interleukin-10 (IL-10), and estrogen receptors [64-fold for estrogen receptor-alpha (ER-α) and 22-fold for ER-beta (ER-β)]. Collectively, data from this latter study suggest that Tregs protect against RV injury through augmented biosynthesis of ERs, COX-2, HO-1, PGI_2,_ and IL-10 in cardiac vascular endothelial cells; this activity points to a homeostatic endothelial cell-Treg interaction.

## An Emerging Rationale for Treg Therapy to Treat PAH

Treg cell therapy holds treatment promise for a variety of conditions (103, 104). Purified Tregs from the patient’s blood are expanded, conditioned, and infused back into the donor’s circulation. Prior studies support the rationale for now developing Treg immunotherapy protocols as a treatment for clinical PAH. This effort will benefit from 1) understanding how infused Tregs home to lungs and draining lymph nodes, 2) discerning how Tregs contribute to vascular repair with more established pulmonary vascular disease, and 3) developing strategies to expand Tregs *ex vivo* while enhancing their activities and stability. Relatively small numbers of Tregs are needed to prevent PAH in rats, but their efficacy with these low numbers diminishes if administered after the disease has progressed (21). Existing literature suggests that the infusion of logarithmically increased numbers of Tregs substantially empowers the treatment effect in established disease (105) and could do so for PAH. More than 50 Treg-infusion clinical trials are being tested for autoimmune and inflammatory diseases (103, 104). Co-medication of IL-2, IL-7, or IL-33 promotes Treg survival and proliferation after cell transfer, and strategies that increase *FOXP3* expression improve Treg persistence and suppressive function (103). Increasing Treg specificity is promoted through genetic engineering that enhances chimeric antigen receptors (CAR) expression in Tregs (106). However, to date, there are no consistent antigen targets for PAH, and polyclonal Treg infusions may be the most feasible starting approach.

## Conclusions

Treg dysfunction is a feature of PAH and contributes to immune dysregulation observed in the disease (9, 107). A unique theme of the research presented in this mini-review is that Tregs can directly afford protection to vascular cells, in addition to its better-studied anti-inflammatory effect on other immune cells. In an athymic rat model treated with SU5416 to induce pulmonary vascular injury, Treg infusion also protected right heart function. Sexual differences are present in various PAH manifestations. Consequently, the study of how regulatory immunity differentially impacts men and women with this condition continues to be an area of promising investigation. Combining immune modulators with vasodilators offers potentially better treatment for PAH. Such an approach was recently taken with a randomized multi-center placebo-controlled clinical trial testing B cell depletion with rituximab to treat systemic sclerosis-associated PAH (108); therapy was safe and potentially effective as an adjunct to standard-of-care vasodilators. For this reason, as Treg therapy becomes a therapeutic option for a variety of immunological disorders (109, 110), vulnerable PAH patients represent a new and promising target population.

## Author Contributions

All authors (WT, SJ, XJ, RT, DK, TG, SA, SP, NV, QT, and MN) contributed to the design, writing and review of this mini review. All authors contributed to the article and approved the submitted version.

## Funding

This work was supported by National Institutes of Health grants HL014985, HL122887, and HL138473 to MN.

## Conflict of Interest

The authors declare that the research was conducted in the absence of any commercial or financial relationships that could be construed as a potential conflict of interest.

## Publisher’s Note

All claims expressed in this article are solely those of the authors and do not necessarily represent those of their affiliated organizations, or those of the publisher, the editors and the reviewers. Any product that may be evaluated in this article, or claim that may be made by its manufacturer, is not guaranteed or endorsed by the publisher.
